# Supplementary material for: The genome sequence of Malacosoma alpicola Staudinger, 1871 (Lepidoptera: Lasiocampidae)
Source: Wellcome Open Res. 2025 Aug 11;10:420. [Version 1] doi: 10.12688/wellcomeopenres.24677.1 (PMC12378395; doi:10.12688/wellcomeopenres.24677.1)
Supplement: Peer review history

# Reviewer report: Reviewer response for version 1

**DOI:** 10.21956/wellcomeopenres.27192.r130452

**Published:** 2025-08-25

**Authors:** Taslima Sheikh

**Affiliations:** 1 Baba Ghulam Shah Badshah University, Rajouri, India

This manuscript represents a scientifically sound and well executed piece of work. The genome assembly is of high quality and the authors have presented the results in a clear and comprehensive manner. No major weaknesses are identified in the rationale, methodology, or presentation. The article meets the necessary standards for indexing and the dataset is expected to be a valuable reference for future research.

**Recommendation**: Accept without revisions.

Are sufficient details of methods and materials provided to allow replication by others?

Yes

Is the rationale for creating the dataset(s) clearly described?

Yes

Are the datasets clearly presented in a useable and accessible format?

Yes

Are the protocols appropriate and is the work technically sound?

Yes

Reviewer Expertise:

lepidoptera

I confirm that I have read this submission and believe that I have an appropriate level of expertise to confirm that it is of an acceptable scientific standard.

# Reviewer report: Reviewer response for version 1

**DOI:** 10.21956/wellcomeopenres.27192.r129371

**Published:** 2025-08-21

**Authors:** Jasmine D. Alqassar

**Affiliations:** 1 The George Washington University, Washington, District of Columbia, USA

In this article Potocký et al. present a high-quality reference genome assembly for the alpine moth *Malacosoma alpicola*. The assembly is highly contiguous, chromosome-level, and complete (BUSCO score: C: 98.2%[S:97.4, D:0.8%], F: 0.3%, M:1.5% n=5286). The inclusion of Merian element assignment to the assembled chromosomes makes this research a valuable resource for studying chromosome evolution in Lepidoptera. The methods are well explained ensuring reproducibility of the study. Additionally the article provides a well-written natural history introduction of this species and rationale for the generation of this data prior to discussion of the genome assembly. This genome resource and accompanying data note will serve as a valuable resource to the research community. 

 The addition of citations that detail the expected genome size and chromosome number for the species, such as cytological evidence if available, or if not available, comparison to genome assemblies of other Lasiocampidae species would strengthen evidence that the assembly is accurate.

Are sufficient details of methods and materials provided to allow replication by others?

Yes

Is the rationale for creating the dataset(s) clearly described?

Yes

Are the datasets clearly presented in a useable and accessible format?

Yes

Are the protocols appropriate and is the work technically sound?

Yes

Reviewer Expertise:

Genomics and evolutionary developmental biology

I confirm that I have read this submission and believe that I have an appropriate level of expertise to confirm that it is of an acceptable scientific standard.
